# A single-dose ChAdOx1-vectored vaccine provides complete protection against Nipah Bangladesh and Malaysia in Syrian golden hamsters

**DOI:** 10.1371/journal.pntd.0007462

**Published:** 2019-06-06

**Authors:** Neeltje van Doremalen, Teresa Lambe, Sarah Sebastian, Trenton Bushmaker, Robert Fischer, Friederike Feldmann, Elaine Haddock, Michael Letko, Victoria A. Avanzato, Ilona Rissanen, Rachel LaCasse, Dana Scott, Thomas A. Bowden, Sarah Gilbert, Vincent Munster

**Affiliations:** 1 Laboratory of Virology, Division of Intramural Research, National Institute of Allergy and Infectious Diseases, National Institutes of Health, Rocky Mountain Laboratories, Hamilton, MT, United States of America; 2 The Jenner Institute, University of Oxford, Oxford, United Kingdom; 3 Rocky Mountain Veterinary Branch, Division of Intramural Research, National Institute of Allergy and Infectious Diseases, National Institutes of Health, Hamilton, MT, United States of America; 4 Division of Structural Biology, Wellcome Centre for Human Genetics, University of Oxford, Oxford, United Kingdom; Pediatric Dengue Vaccine Initiative, UNITED STATES

## Abstract

Nipah virus (NiV) is a highly pathogenic re-emerging virus that causes outbreaks in South East Asia. Currently, no approved and licensed vaccine or antivirals exist. Here, we investigated the efficacy of ChAdOx1 NiV_B_, a simian adenovirus-based vaccine encoding NiV glycoprotein (G) Bangladesh, in Syrian hamsters. Prime-only as well as prime-boost vaccination resulted in uniform protection against a lethal challenge with NiV Bangladesh: all animals survived challenge and we were unable to find infectious virus either in oral swabs, lung or brain tissue. Furthermore, no pathological lung damage was observed. A single-dose of ChAdOx1 NiV_B_ also prevented disease and lethality from heterologous challenge with NiV Malaysia. While we were unable to detect infectious virus in swabs or tissue of animals challenged with the heterologous strain, a very limited amount of viral RNA could be found in lung tissue by *in situ* hybridization. A single dose of ChAdOx1 NiV_B_ also provided partial protection against Hendra virus and passive transfer of antibodies elicited by ChAdOx1 NiV_B_ vaccination partially protected Syrian hamsters against NiV Bangladesh. From these data, we conclude that ChAdOx1 NiV_B_ is a suitable candidate for further NiV vaccine pre-clinical development.

## Author summary

Nipah virus was discovered in 1998 after an outbreak in Malaysia. Since then, several outbreaks have been reported in Bangladesh and India. Although most outbreaks are relatively small, a very high case-fatality rate is reported (75%). Furthermore, human-to-human transmission has been reported. Currently, no approved vaccine or countermeasure exist. In this manuscript, we discuss a vaccine based on a chimpanzee adenovirus. Importantly, the vaccine vector (ChAdOx1) is in clinical trials. In the work presented here, we show that this vaccine is fully protective against both genotypes of Nipah virus. Furthermore, we observe partial protection against Hendra virus, a related virus. Antibodies produced upon vaccination with our vaccine alone are partially protective against Nipah virus. This is an important step forwards towards the development of an approved vaccine for Nipah virus.

## Introduction

Nipah virus (NiV) is a highly pathogenic emerging virus in the family *Paramyxoviridae*, genus *Henipavirus*. The virus was first detected in 1998 when it caused an outbreak of severe, rapidly progressing encephalitis in pig farmers in Malaysia and Singapore, with a case-fatality rate of 38% [1]. Pigs were most likely infected after eating fruit contaminated by infected fruit bats of the genus *Pteropus*, the animal reservoir of NiV [2]. This was then followed by pig-to-human infection, but only very limited human-to-human transmission was reported [3]. Since then, NiV has caused near annual outbreaks in Bangladesh and India. Reported outbreaks in these countries involve fewer patients, with a higher case-fatality rate (average 75%) [4] and, in contrast to the Malaysia outbreak, there are well-documented cases of human-to-human transmission [5]. It has been hypothesized that the source for NiV infection in index cases in India and Bangladesh is palm sap contaminated with bat urine [6–9]. The most recent outbreak of NiV occurred in the Indian state Kerala in May 2018 where 19 patients were infected resulting in 17 fatalities. Although this state had not seen NiV infections before 2018, bats of the genus *Pteropus* are prevalent in this area. Sequence analyses have demonstrated that isolates originating from Malaysia and Bangladesh represent two different genetic lineages [10–12].

A second member of the *Henipavirus* genus is Hendra virus (HeV), which is characterized by a similar pathology and has caused infections in humans in Australia [13].

NiV-caused disease is characterized by the onset of non-specific symptoms such as fever, headache, dizziness, and myalgia. Hereafter patients may develop severe encephalitis and pulmonary disease. Pulmonary disease is observed more frequently in patients infected with NiV Bangladesh. A unique potential complication is the late onset or relapsing encephalitis, which has been documented up to 11 years after NiV infection [14].

The host range of NiV is broad, facilitated by the use of the conserved ephrin-B2 and–B3 as cellular receptors [15], raising the possibility of further outbreaks resulting from transmission from infected livestock or domestic animals. The current lack of licensed vaccines or treatments has prompted the WHO to identify NiV as a pathogen requiring urgent investment into development of countermeasures [16]. Given the sporadic nature of NiV outbreaks, the aim is to develop a vaccine that demonstrates protective efficacy in animal models and acceptable safety and immunogenicity profiles in phase I and II clinical trials. Vaccines which meet these criteria will be stockpiled and may then be used in the event of an outbreak, following clinical trial protocols for use prepared in advance.

Apart from demonstrating efficacy against challenge in animal models, other desirable characteristics for a vaccine to be stockpiled are the availability of large-scale manufacturing processes, thermostability, and safety in all sections of the population including the youngest, oldest, and immunocompromised patients [17].

ChAdOx1-vectored vaccines fulfil all these requirements, making this a promising platform. The ChAdOx1 vector is a replication-deficient simian adenovirus vector which has been used to produce several vaccines which are now in clinical development. A common feature of these vaccines is their low reactogenicity, strong immunogenicity, and the absence of vector replication after immunization, an important safety feature. In pre-clinical studies a single dose of ChAdOx1 vectored vaccines has been shown to be protective against infection with Rift Valley Fever Virus, Middle East respiratory syndrome coronavirus, *Mycobacterium tuberculosis* and Zika virus [18–21]. Large scale manufacturing has been performed for replication-deficient adenoviral vectored vaccines for Ebola, with one vaccine now licensed and another in advanced clinical development [22, 23]. Further, a simple thermostabilization process allows for vaccine storage at ambient temperatures [24], removing the need for a cold chain for storage and shipping. We now report on pre-clinical immunogenicity and efficacy testing of ChAdOx1 NiV_B_.

## Materials and methods

### Ethics statement

Animal experiment approval was received from the Institutional Animal Care and Use Committee (IACUC) at Rocky Mountain Laboratories. Experiments were performed in an Association for Assessment and Accreditation of Laboratory Animal Care-approved facility by certified staff, following the guidelines and basic principles in the NIH Guide for the Care and Use of Laboratory Animals, the Animal Welfare Act, United States Department of Agriculture and the United States Public Health Service Policy on Humane Care and Use of Laboratory Animals (Protocol # 2017-033E and 2018-035E). The Institutional Biosafety Committee (IBC) approved work with infectious NiV and Hendra virus (HeV) strains under BSL4 conditions and sample inactivation was performed according to IBC-approved standard operating procedures for removal of specimens from high containment.

### Cells and virus

Henipavirus isolates were obtained from the Special Pathogens Branch of the Centers for Disease Control and Prevention, Atlanta, GA or Public Health Agency, Winnipeg, Canada. NiV Bangladesh (GenBank no. AY988601), NiV Malaysia (GenBank no. AF212302), and HeV (GenBank no. AF017149) have been passaged three, four, and three times in VeroE6 cells respectively. All virus propagation in this manuscript was performed in VeroE6 cells in Dulbecco’s modified Eagle’s medium (DMEM, Sigma) supplemented with 2% fetal bovine serum (Gibco), 1 mM L-glutamine (Gibco), 50 U/ml penicillin (Gibco), and 50 μg/ml streptomycin (Gibco) (2% DMEM). VeroE6 cells were maintained in DMEM supplemented with 10% fetal bovine serum, 1 mM L glutamine, 50 U/ml penicillin and 50 μg/ml streptomycin.

### ChAdOx1 NiV_B_ generation and production

The glycoprotein (G) gene from Nipah virus (Bangladesh outbreak 2008–2010, Genbank accession number: JN808864.1) was codon optimized for humans and synthesized by GeneArt (Thermo Fisher Scientific‎). The synthesized G gene was cloned into a transgene expression plasmid comprising a modified human cytomegalovirus immediate early promoter (CMV promoter) with tetracycline operator (TetO) sites and the polyadenylation signal from bovine growth hormone (BGH). The resulting expression cassette was inserted into the E1 locus of a genomic clone of ChAdOx1 using site-specific recombination [25]. The virus was rescued and propagated in T-REx-293 cells (Invitrogen). Purification was by CsCl gradient ultracentrifugation, and the virus was titered as previously described [26]. Doses for vaccination were based on infectious units (IU).

### Vaccination studies

Female Golden Syrian hamsters (4–6 weeks old) were purchased from Envigo. Animals were vaccinated I.M. with 50 μl of 10^8^ IU of vaccine or injected I.M. with 50 μl of saline, in each thigh (100 μl total volume). For the homologous challenge vaccine experiment, animals were vaccinated at D-70 and/or D-42. For the heterologous challenge experiment, animals were vaccinated at D-28. Three days prior to vaccination and virus challenge animals were bled via orbital sinus puncture. All animals were challenged with 1000LD_50_ of virus in 500 μl DMEM via I.P. inoculation: NiV Bangladesh = 5.3 x 10^5^ TCID_50_; NiV Malaysia = 6.8 x 10^4^ TCID_50_; HeV = 6.0 x 10^3^ TCID_50_. We chose the I.P. route as a uniformly lethal challenge route and to be able to compare with previously conducted vaccine experiments [27]. For each study group, 10 hamsters were utilized. Of these, four animals were euthanized 4 (HeV) or 5 (NiV) days post inoculation and the remaining six animals were followed for 28 days post challenge. Weight was recorded daily up to 10 days post infection, and oropharyngeal swabs were taken daily up to 7 days post inoculation in 1 mL of DMEM. Animals were euthanized when >20% of weight loss was recorded, or severe disease signs (e.g. difficulty breathing or paralysis) were observed. Upon euthanasia, blood and tissues were collected and subsequently analyzed for virology and histology as approved by IACUC.

### Passive transfer study

Female Golden Syrian hamsters (4–6 weeks old) were purchased from Envigo. Fifteen animals were vaccinated with either ChAdOx1 NiV_B_ or ChAdOx1 GFP as described above at 56 and 28 days before serum collection. Serum was collected via cardiac puncture, pooled per vaccine group and IgGs were purified using the MAbtrap kit (Sigma) according to manufacturer’s instructions from 10 mL of serum. Purified IgGs were filtered through an 0.45μm filter and diluted to 4.5 mL in sterile PBS. Ten hamsters were immunized via I.P. injection using 400 μl per hamster. All animals were challenged as described above one day post treatment. For each study group, 10 hamsters were utilized. Of these, four animals were euthanized 5 days post challenge and the remaining six animals were followed for 56 days post challenge. Weight was recorded daily up to 10 days post challenge, and oropharyngeal swabs were taken daily up to 7 days post inoculation in 1 mL of DMEM. Animals were euthanized when >20% of weight loss was recorded, or severe disease signs (e.g. difficulty breathing or paralysis) were observed. Upon euthanasia, blood and tissues were collected and subsequently analyzed for virology and histology as approved by IACUC.

### Titration assay

Virus titrations were performed by end-point titration in VeroE6 cells, which were inoculated with tenfold serial dilutions of virus swab media or tissue homogenates. After 1hr incubation at 37°C and 5% CO_2_, tissue homogenate dilutions were removed, washed twice with PBS and replaced with 100 μl 2% DMEM. Cytopathic effect was scored at 5 dpi and the TCID_50_ was calculated from 4 replicates by the Spearman-Karber method [28].

### Virus neutralization assay

Sera was heat-inactivated (30 min, 56°C) and two-fold serial dilutions were prepared in 2% DMEM. Hereafter, 100 TCID_50_ of NiV was added. After 1hr incubation at 37°C, virus was added to VeroE6 cells and incubated at 37°C and 5% CO_2_. At 5 dpi, cytopathic effect was scored. The virus neutralization titer was expressed as the reciprocal value of the highest dilution of the serum which still inhibited virus replication.

### Production NiV G and F proteins

NiV-G Malaysia (residues E144—T602, gene accession number NC_002728) was cloned into the pHLSEC mammalian expression vector [29] and NiV-F Malaysia (residues G26—D482, gene accession number AY816748.1) was cloned into the pHLSEC vector containing a C-terminal GCNt trimerization motif [30]. The constructs were transiently expressed in human embryonic kidney (HEK) 293T cells in roller bottles, as described previously [29]. Supernatant was harvested 96 hours after transfection and diafiltrated using the AKTA FLUX system (GE Healthcare) against either PBS, pH 7.4 (NiV-G) or buffer containing 10 mM Tris and 150 mM NaCl, pH 8.0 (NiV-F). The proteins were further purified by Ni-NTA immobilized metal-affinity chromatography using His-Trap HP columns (GE Healthcare) followed by size exclusion chromatography. NiV-G was purified using a Superdex 200 10/300 Increase GL column (GE healthcare) equilibrated in PBS pH 7.4 and NiV-F was purified using a Superose 6 Increase 10/300 GL column (GE healthcare) equilibrated in 10 mM Tris and 150 mM NaCl pH 8.0.

### ELISA

Maxisorp plates (Nunc) were coated overnight at 4°C with 5 μg of G or F protein per plate in Carb/Bicarb binding buffer (4.41 g KHCO_3_ and 0.75 g Na_2_CO_3_ in 1 L distilled water). After blocking with 5% milk in PBS with 0.01% tween (PBST), serum (2x serial diluted starting at 100x dilution) in 5% milk in PBST was incubated at RT for 1 hr. Antibodies were detected using affinity-purified antibody peroxidase-labeled goat-anti-hamster IgG (Fisher, 14-22-06) in 5% milk in PBST and TMB 2-component peroxidase substrate (Seracare) and read at 450 nm. All wells were washed 3x with PBST in between steps. Prior to using F and G proteins based on NiV Malaysia, we established that cross-reactivity with NiV Bangladesh antibodies was sufficient for usage in ELISA by testing sera known to be positive for NiV Bangladesh antibodies.

### Histology and in situ hybridization

Necropsies and tissue sampling were performed according to IBC-approved protocols. Harvested tissues were fixed for a minimum of 7 days in 10% neutral-buffered formalin and subsequently embedded in paraffin. Hematoxylin and eosin (H&E) staining and in situ hybridization (ISH) were performed on tissue sections and cell blocks. Detection of NiV and HeV viral RNA was performed using the RNAscope FFPE assay (Advanced Cell Diagnostics Inc., Newark, USA) as previously described [31] and in accordance with the manufacturer’s instructions. Briefly, tissue sections were deparaffinized and pretreated with heat and protease before hybridization with target-specific probes for NiV or HeV. Ubiquitin C and the bacterial gene, dapB, were used as positive and negative controls, respectively. Whole-tissue sections for selected cases were stained for NiV and HeV viral RNA, UBC and dapB by the RNAscope VS FFPE assay (RNAscopeVS, Newark, USA) using the Ventana Discovery XT slide autostaining system (Ventana Medical Systems Inc., Tucson, USA). A board-certified veterinary anatomic pathologist evaluated all tissue slides.

### Statistical analysis

Statistical analysis was performed by the Log-rank (Mantel-Cox) test to compare survival curves, and by Welch-corrected one-tailed unpaired student’s t-test to compare infectious virus titers in tissue. SEM was calculated for all samples. *P*-values < 0.05 were significant.

## Results

### Homologous challenge with NiV Bangladesh of Syrian Golden hamsters vaccinated with ChAdOx1 NiV_B_

**Fig 1:**
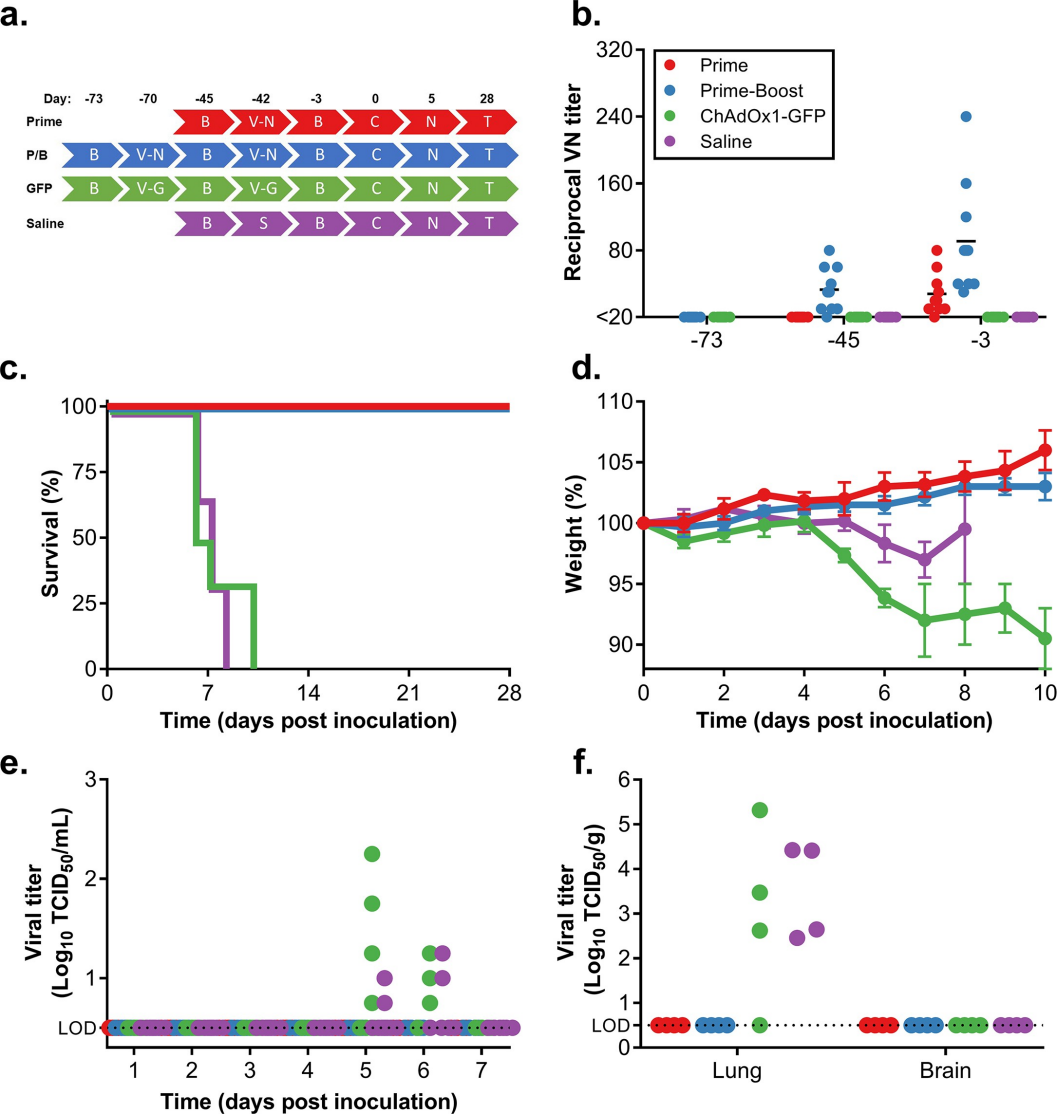
Single-dose vaccination with ChAdOx1 NiV_B_ protects Syrian hamsters against a lethal challenge with NiV Bangladesh. (a) Experimental layout. P/B = prime-boost; B = bleed; V-N = ChAdOx1 NiV_B_ vaccination; V-G = ChAdOx1 GFP injection; S = saline injection; C = challenge; N = necropsy; T = Termination experiment. (b) Neutralizing antibodies in serum were determined via virus neutralization assay on VeroE6 cells at D-73, D-45 and D-3. (c) Survival of Syrian hamsters challenged with NiV Bangladesh. Survival of vaccinated animals was significant compared to control animals (*P* = <0.0001). (d) Weight loss of challenged animals. (e) Shedding of infectious virus in oropharyngeal swabs collected daily from challenged animals. NiV titers were determined via endpoint titration on VeroE6 cells. (f) Infectious virus titers in lung and brain tissue collected from challenged animals at D5. NiV titers were determined via endpoint titration on VeroE6 cells.

To determine efficacy of the ChAdOx1 NiV_B_ vaccine, we vaccinated groups of 10 hamsters with either a single dose at D-42 or a prime-boost regime at D-70 and D-42. As control groups, we either injected hamsters with ChAdOx1 GFP at D-70 and D-42 or saline at D-42 (*Fig 1A*). Virus neutralizing antibodies could be detected after a single dose of ChAdOx1 NiV_B_ and increased upon a secondary dose (average VN titer ± SEM = 30.5 ± 5.7 after single dose, 91 ± 21 after boost). In contrast, no neutralizing antibodies could be detected in serum obtained from the control groups (*Fig 1B*). All hamsters were challenged with a lethal dose of NiV Bangladesh (1000 LD_50_) via intraperitoneal inoculation on D0 (*Fig 1A*). All vaccinated animals survived challenge and did not show signs of disease, such as weight loss, at any stage throughout the experiment. This was in contrast to the control groups in which all animals succumbed to disease between D6 and D10 and exhibited weight loss (*Fig 1C and 1D*), as well as respiratory and/or neurological signs, including labored breathing and paralyzed hind legs. Statistical analysis demonstrated that survival in the vaccinated groups was significant compared to both control groups (P < 0.0001).

Oropharyngeal swabs were taken daily and assessed for infectious virus by limiting dilution titrations. None of the vaccinated animals shed virus at any timepoint. In contrast, control animals from both groups were found to shed virus at D5 and D6 (*Fig 1E*).

Four animals of each group were euthanized at D5 and lung and brain tissue were harvested. Infectious virus could only be detected in lung tissue of animals from both control groups (average titer ± SEM = 3.3 x 10^4^ ± 2.5 x 10^4^ TCID_50_/g of tissue) and was not detected in any tissue of the vaccinated animals (*Fig 1F*). We did not observe any differences between the two control groups.

**Fig 2:**
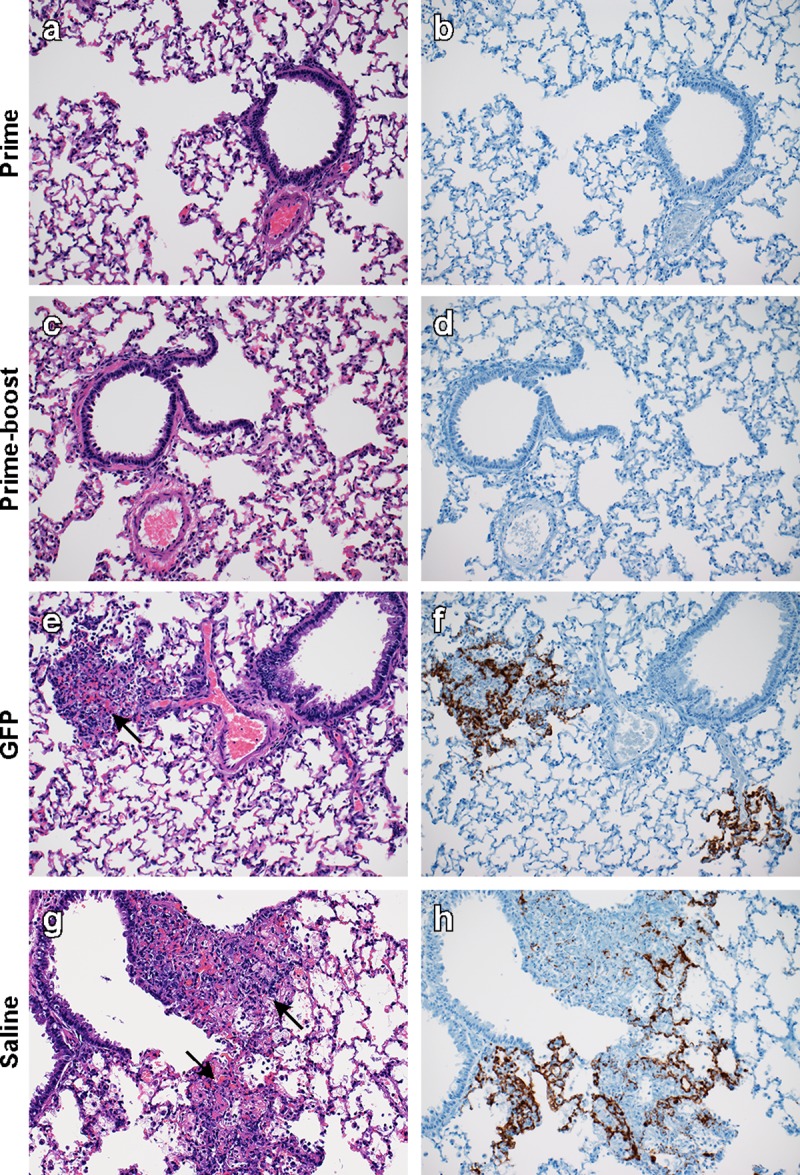
Single-dose vaccination with ChAdOx1 NiV_B_ protects Syrian hamsters against bronchointerstitial pneumonia caused by NiV Bangladesh challenge. Hamsters were vaccinated with ChAdOx1 NiV_B_ or injected with ChAdOx1 FGP or saline and challenged with NiV Bangladesh. Lung tissue was collected at D5. Tissue sections were stained with hematoxylin-eosin (left panels) or with target-specific probes for NiV RNA, which is visible as a red-brown staining (right panels). (a-b) Lung tissue obtained at D5 of animals vaccinated with a single-dose of ChAdOx1 NiV_B_. (c-d) Lung tissue obtained at D5 of animals vaccinated with a prime-boost regime of ChAdOx1 NiV_B_. (e-f) Lung tissue obtained at D5 of animals injected with a prime-boost regime of ChAdOx1 FGP. (g-h) Lung tissue obtained at D5 of animals injected with saline. Arrow = Inflammatory cells, fibrin, edema and hemorrhage centered on terminal bronchioles. 200x magnification. H&E and ISH slides were from sequential sections.

Lung and brain tissue harvested at D5 were then evaluated for pathological changes. None of the vaccinated animals displayed pulmonary pathology and no viral RNA was detected in lung tissue by ISH. Control animals developed pulmonary lesions that were indistinguishable between the two groups. These hamsters developed bronchointerstitial pneumonia that was characterized by multifocal inflammatory nodules that were centered on terminal bronchioles and extend into adjacent alveoli. The nodules were composed of large numbers of foamy macrophages and fewer neutrophils and lymphocytes admixed with small amounts of necrotic debris. In most cases hemorrhage, fibrin and edema admixed with inflammatory cells was observed. Edema and fibrin often were extended into surrounding alveoli. Alveoli that were adjacent to areas of inflammation were thickened by fibrin, edema and small numbers of macrophages and neutrophils as previously observed in NiV infected hamsters [32]. There was abundant viral RNA demonstrated by ISH in areas of inflammation (brown staining). The viral RNA was predominantly found in type I pneumocytes but was also multifocally present in vascular and bronchiolar smooth muscle and endothelial cells (*Fig 2*).

### Heterologous challenge with NiV Malaysia or HeV of Syrian Golden hamsters vaccinated with ChAdOx1 NiV_B_

**Fig 3:**
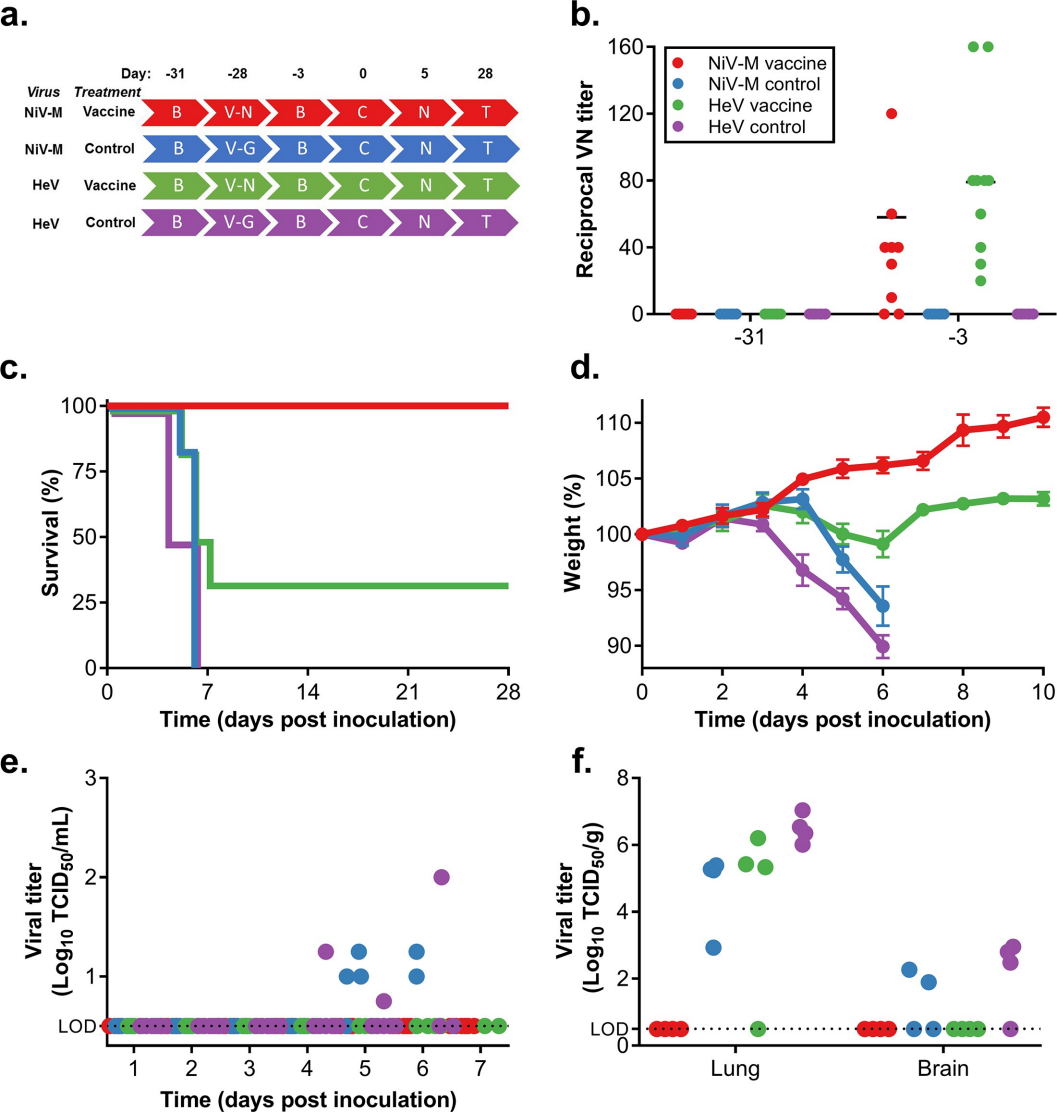
Single-dose vaccination with ChAdOx1 NiV_B_ protects Syrian hamsters against a lethal challenge with NiV Malaysia, but not HeV. (a) Experimental layout. B = bleed; V-N = ChAdOx1 NiV_B_ vaccination; V-G = ChAdOx1 FGP injection; C = challenge; N = necropsy; T = Termination experiment. (b) Neutralizing antibodies in serum were determined via virus neutralization assay on VeroE6 cells at D-31 and D-3. (c) Survival of Syrian hamsters challenged with NiV Malaysia or HeV. (d) Weight loss of challenged animals. (e) Shedding of infectious virus in oropharyngeal swabs collected daily from challenged animals. Virus titers were determined via endpoint titration on VeroE6 cells. (f) Infectious virus titers in lung and brain tissue collected from challenged animals at D4 (HeV) or D5 (NiV). Virus titers were determined via endpoint titration on VeroE6 cells.

To determine efficacy of ChAdOx1 NiV_B_ against NiV Malaysia and HeV, groups of 10 hamsters were vaccinated with a single dose of ChAdOx1 NiV_B_ or a single dose of ChAdOx1 GFP at D-28 (*Fig 3A*). As before, virus neutralizing antibodies could be detected after vaccination with ChAdOx1 NiV_B_ but not upon injection with ChAdOx1 GFP (Average VN titer ± SEM = 68.6 ± 13.6) (*Fig 3B*). Subsequently, hamsters were challenged with either NiV Malaysia or HeV (1000 LD_50_) via intraperitoneal inoculation on D0 (*Fig 3A*). All vaccinated animals challenged with NiV Malaysia survived with no signs of disease such as weight loss at any stage throughout the experiment. In contrast, animals challenged with NiV Malaysia that received ChAdOx1 FGP all succumbed to infection between D5 and D6. These animals experienced weight loss and respiratory and neurological signs (*Fig 3C and 3D*). Statistical analysis demonstrated that survival in the vaccinated group was significantly different from the control group (*P*  =  0.0012).

Oropharyngeal swabs were taken daily and assessed for infectious virus. None of the vaccinated animals challenged with NiV Malaysia shed virus at any timepoint. In contrast, control animals challenged with NiV Malaysia were found to shed virus at D5 and D6 (*Fig 3E*).

Four animals from both groups were euthanized at D5 and lung and brain tissue were harvested. Infectious virus could only be detected in lung and brain tissue of animals from the control group (average virus titer lung ± SEM = 1.5 x 10^5^ ± 5.2 x 10^4^ TCID_50_/g, brain ± SEM = 6.8 x 10^1^ ± 4.4 x 10^1^ TCID_50_/g) and was not detected in any tissue of the vaccinated animals (*Fig 3F*).

Four out of six vaccinated animals challenged with HeV succumbed to disease between D5 and D7. The two survivors showed minimal weight loss (<2%) and no signs of disease. Animals that received ChAdOx1 FGP all succumbed to HeV infection between D4 and D6. These animals showed weight loss as well as respiratory and neurological signs (*Fig 3C and 3D*). Log-rank (Mantel-Cox) test demonstrated that survival in the vaccinated group was significant (*P*  =  0.0476) compared to the control group.

Oropharyngeal swabs were taken daily and assessed for infectious virus. None of the vaccinated animals challenged with HeV shed virus at any timepoint. In contrast, control animals challenged with HeV were found to shed virus at D4, D5 and D6 (*Fig 3E*).

Four animals from both groups were euthanized at D4 and lung and brain tissue were harvested. Infectious virus was detected in three out of four lungs of the vaccinated animals and all lungs of the control animals (average virus titer ± SEM = 5.2 x 10^5^ ± 3.6 x 10^5^ and 4.4 x 10^6^ ± 2.2 x 10^6^ TCID_50_/g tissue for vaccinated and control animals, respectively). No statistical difference in infectious virus titer was found between the two groups using an unpaired one-tailed Student’s t-test (*P* = 0.0674). Infectious virus was only detected in brain tissue of animals from the control group (average titer ± SEM = 4.6 x 10^2^ ± 2.0 x 10^2^ TCID_50_/g) and not in vaccinated animals (*Fig 3F*).

**Fig 4:**
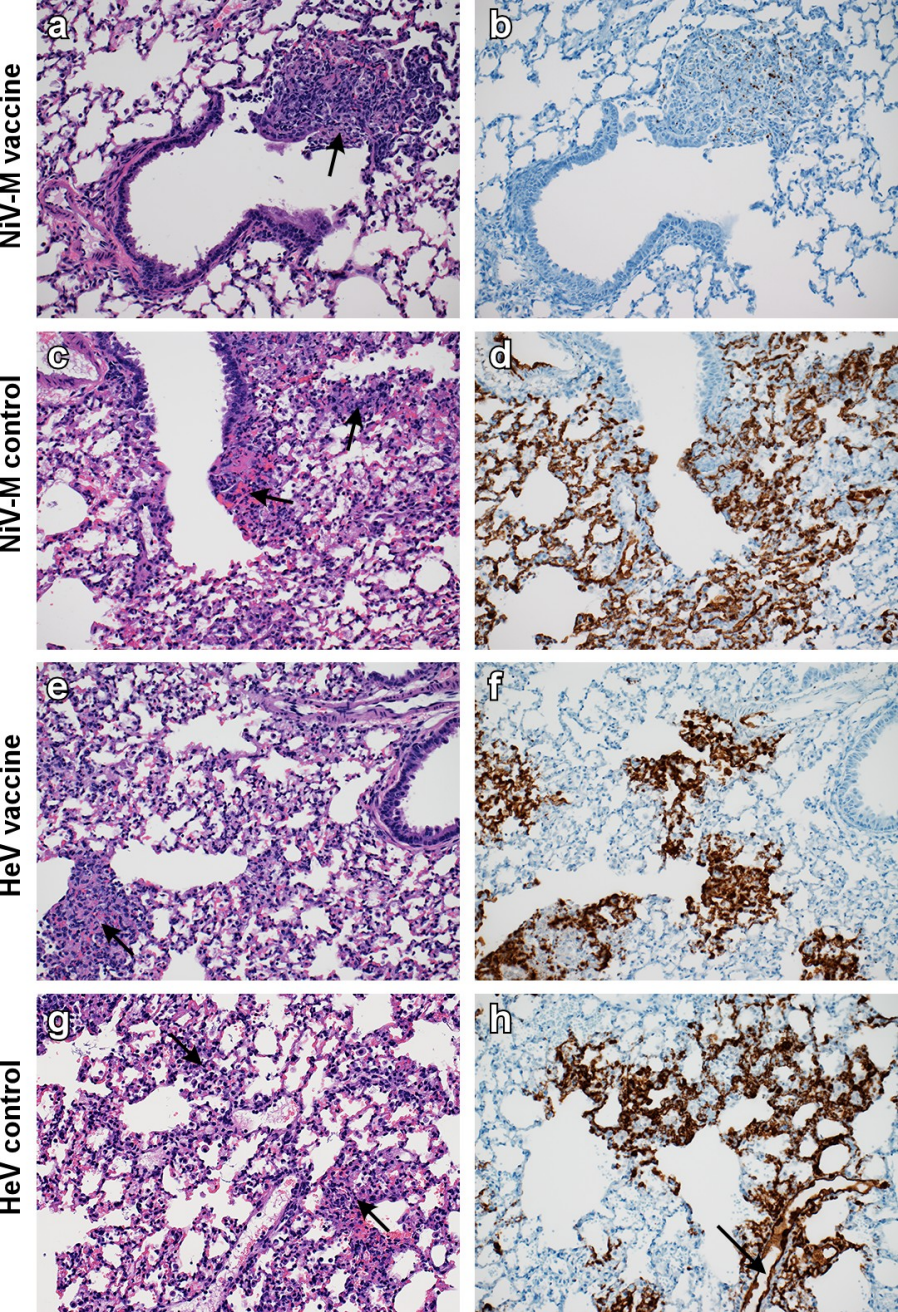
Single-dose vaccination with ChAdOx1 NiV_B_ protects Syrian hamsters against severe bronchointerstitial pneumonia caused by NiV Malaysia challenge, but not by HeV challenge. Hamsters were vaccinated with ChAdOx1 NiV_B_ or injected with ChAdOx1 FGP and challenged with NiV Malaysia or HeV. Lung tissue was collected at D4 (HeV) or D5 (NiV). Tissue sections were stained with hematoxylin-eosin (left panels) or with target-specific probes for viral RNA, which is visible as a red-brown staining (right panels). (a-b) Lung tissue obtained at D5 of animals vaccinated with a single-dose of ChAdOx1 NiV_B_ and challenged with NiV Malaysia. (c-d) Lung tissue obtained at D5 of animals injected with a single-dose of ChAdOx1 FGP and challenged with NiV Malaysia. (e-f) Lung tissue obtained at D5 of animals vaccinated with a single-dose of ChAdOx1 NiV_B_ and challenged with HeV. (g-h) Lung tissue obtained at D5 of animals injected with a single-dose of ChAdOx1 FGP and challenged with HeV. Arrow in H&E = Inflammatory cells, fibrin, edema and hemorrhage centered on terminal bronchioles. Arrow in ISH = Positive endothelial cells lining an artery. 200x magnification. H&E and ISH slides were from sequential sections.

Harvested lung tissue was then evaluated for pathological changes. All four groups of hamsters developed pulmonary lesions. All animals challenged with HeV and control animals challenged with NiV Malaysia developed bronchointerstitial pneumonia which was indistinguishable from the lesions described for the control animals in the homologous challenge study. Vaccinated hamsters challenged with NiV Malaysia developed mild to moderate bronchointerstitial pneumonia and did not display any evidence of pulmonary edema, fibrin or hemorrhage. ISH demonstrated viral RNA predominantly in type I pneumocytes and rarely in vascular and bronchiolar smooth muscle and endothelial cells in animals challenged with HeV and control animals challenged with NiV Malaysia. In vaccinated animals challenged with NiV Malaysia, however; there was very little RNA present and only in type I pneumocytes in areas of inflammation (*Fig 4*).

### Protective efficacy of antibodies elicited by ChAdOx1 NiV_B_ in Syrian hamsters

**Fig 5:**
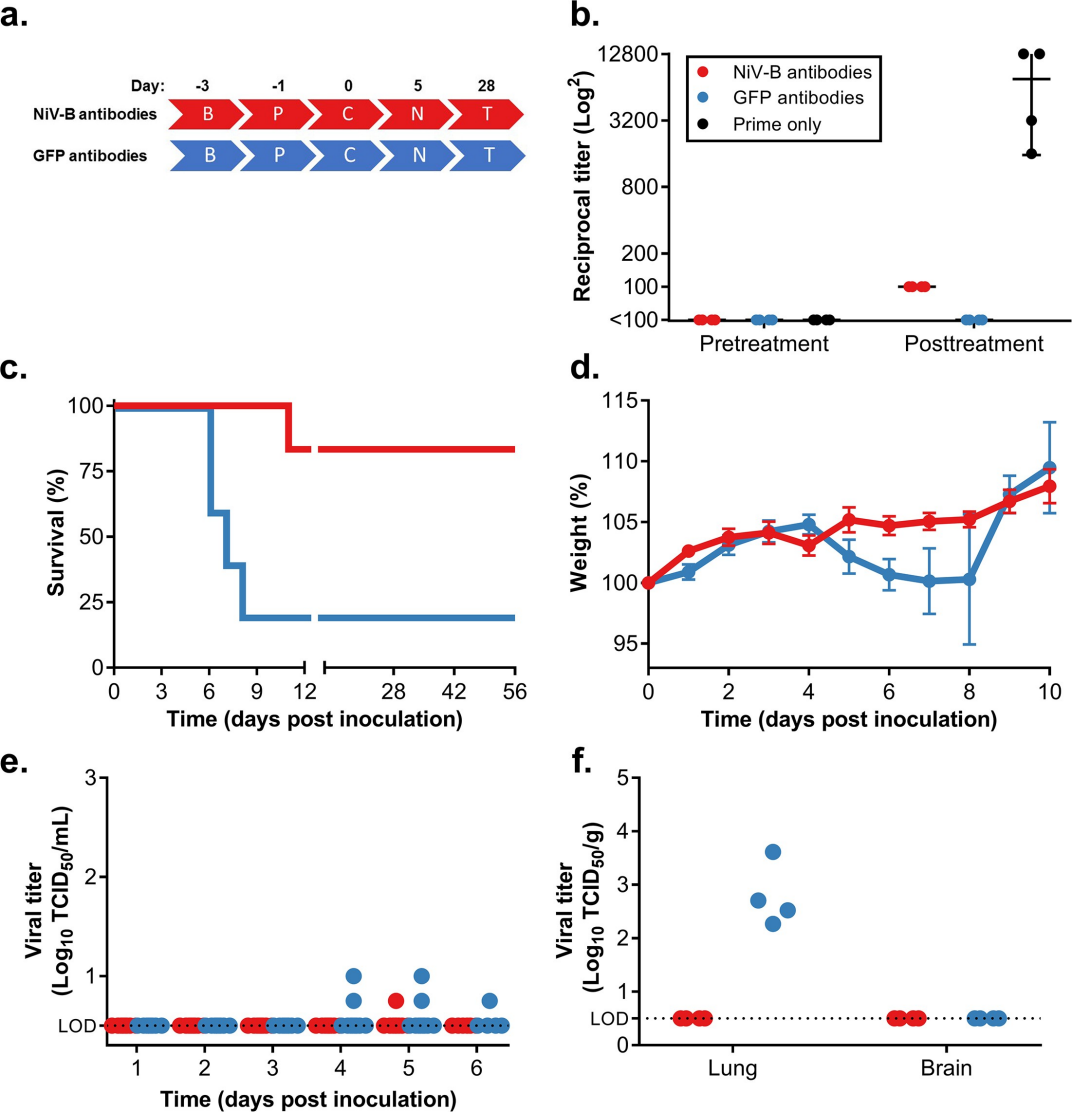
Treatment with ChAdOx1 NiV_B_ elicited antibodies provides partial protection against a lethal NiV Bangladesh challenge in Syrian hamsters. (a) Experimental layout. B = bleed; P = passive transfer; C = challenge; N = necropsy; T = Termination experiment. (b) Reciprocal titer of NiV Bangladesh G protein-specific antibodies in serum as determined via ELISA at pre-treatment and post-treatment. As a control, serum obtained from animals vaccinated via prime only (*Fig* 1) were taken along. (c) Survival of animals challenged with NiV Bangladesh treated with NiV or GFP antibodies. (d) Weight loss of challenged animals. (e) Shedding of infectious virus in oropharyngeal swabs collected daily from challenged animals. (f) Infectious virus titers in lung and brain tissue collected from challenged animals at D5. Virus titers were determined via endpoint titration on VeroE6 cells.

Finally, we wanted to assess the protective effect of antibodies elicited after ChAdOx1 NiV_B_ vaccination. Two groups of 15 hamsters were either vaccinated with ChAdOx1 NiV_B_ or injected with ChAdOx1 FGP at D-56 and D-28. All animals were bled at D0 and we collected 13 and 15 mL respectively. IgG was purified from 10 mL pooled serum. Ten animals per group were then injected peritoneally with purified IgG. Animals were challenged with a lethal dose of NiV Bangladesh (1000 LD_50_) one day post passive transfer (*Fig 5A*). We were unable to detect neutralizing antibodies in serum obtained at D5 from four hamsters from each group. However, serum from animals treated with NiV antibodies was positive by ELISA against NiV G protein, albeit with a lower reciprocal titer than antibodies in serum obtained from single-dose vaccinated animals (*Fig 5B*). One out of six animals treated with NiV antibodies succumbed to disease on D11. No weight loss was observed, however the animal showed severe neurological signs. None of the other NiV antibody-treated animals experienced weight loss or signs of disease. Four out of six animals treated with GFP antibodies succumbed to disease between D6 and D8. These animals showed weight loss and respiratory or neurological signs. The two surviving animals did not show any signs of disease throughout the experiment. One of these animals did not seroconvert as measured by ELISA against NiV F and G protein, and it was suspected this animal was not infected. Therefore, this animal was excluded from the survival curve. The log-rank (Mantel-Cox) test demonstrated that survival in the treated group was significant (*P*  =  0.0168) compared to the control group (*Fig 5C and 5D*).

Oropharyngeal swabs were taken daily and assessed for infectious virus. Shedding was minimal and found in one animal treated with NiV antibodies on D5, and five animals treated with GFP antibodies between D4 and D6 (*Fig 5E*).

Four animals from both groups were euthanized at D5 and lung and brain tissue were harvested. Infectious virus could only be detected in lung tissue of animals treated with GFP antibodies and was not detected in any tissue of the animals treated with NiV antibodies (*Fig 5F*).

**Fig 6:**
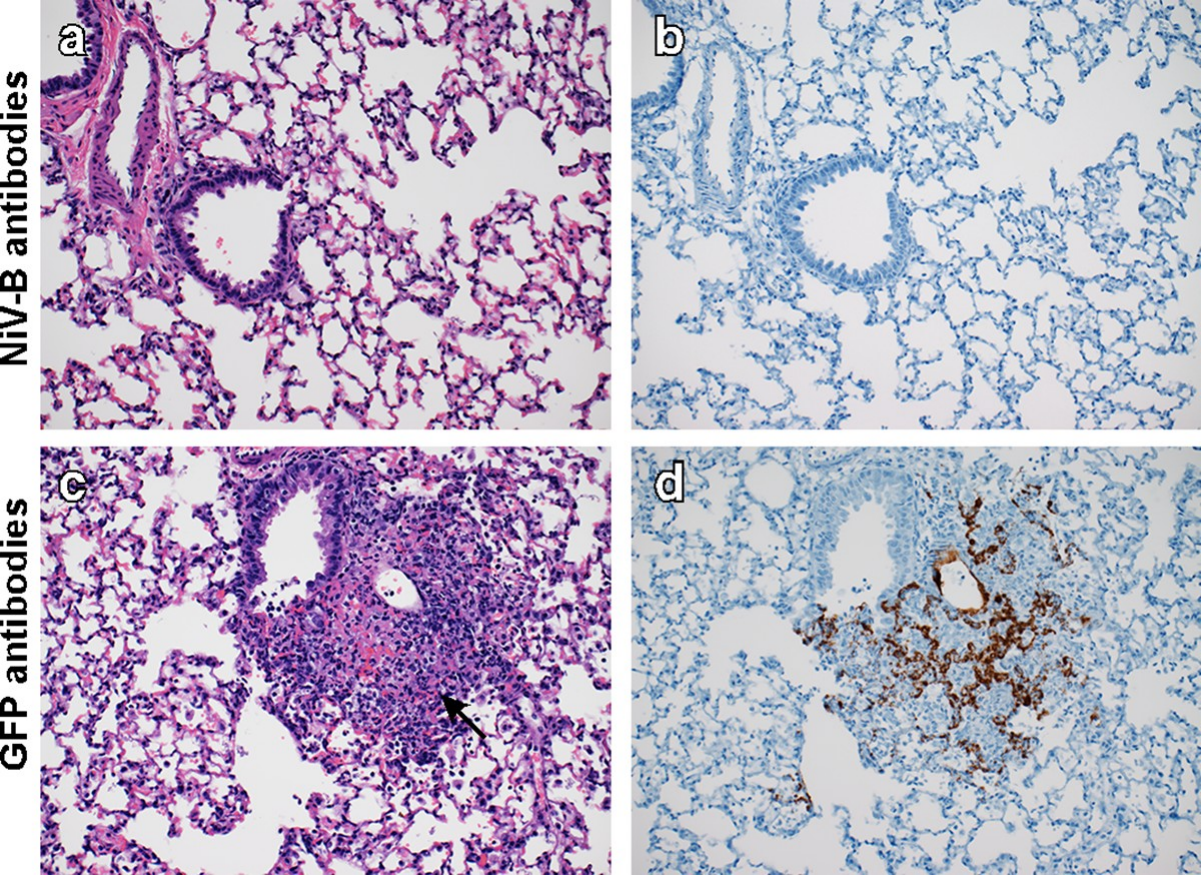
Treatment with IgG antibodies elicited by ChAdOx1 NiV_B_ vaccination reduced pathology and viral RNA abundance in Syrian hamsters challenged with NiV Bangladesh. Hamsters were treated with NiV or GFP antibodies and challenged with NiV Bangladesh. Lung tissue was collected at D5. Tissue sections were stained with hematoxylin-eosin (left panels) or with target-specific probes for viral RNA, which is visible as a brown staining (right panels). (a-b) Lung tissue obtained at D5 of animals treated with NiV antibodies. (c-d) Lung tissue obtained at D5 of animals treated with GFP antibodies. Arrow in H&E = Inflammatory cells, fibrin, edema and hemorrhage centered on terminal bronchioles. 200x magnification. H&E and ISH slides were from sequential sections.

Lung tissue harvested at D5 was then evaluated for pathological changes. Both groups of hamsters developed pulmonary lesions similar to those described in the homologous challenge study, however; the NiV antibody-treated hamsters developed mild to moderate pulmonary lesions whereas the control animals developed severe lesions. Additionally, none of the NiV antibody-treated hamsters displayed any pulmonary fibrin, edema or hemorrhage. ISH demonstrated viral RNA in type I pneumocytes in areas of inflammation. Abundance of viral RNA was notably less in animals treated with NiV antibodies (*Fig 6*).

## Discussion

NiV is a re-emerging infectious disease which causes outbreaks with a high case-fatality rate.

No licensed vaccine against NiV currently exists, and it is therefore key that a safe and effective vaccine be developed. Several vaccine candidates have been explored in different animal models. These can be categorized as subunit vaccines or live-vectored vaccines that target the NiV outer membrane proteins G and/or F. Protection against disease and lethality has been shown in hamsters [27, 33], pigs [34, 35], African green monkeys [36–38], cats [39], and ferrets [40, 41]. Efficacy is thought to be mediated by neutralizing antibodies, as passive transfer of antibodies in naive animals also results in protection against disease [27, 42]. These approaches are promising, but no vaccine candidates have so far been moved into clinical trials.

In the studies presented here, we tested the efficacy of a vaccine based on NiV Bangladesh G protein in a replication-deficient simian adenovirus vector in Syrian hamsters. A prime-only as well as a prime-boost regime protected Syrian hamsters against challenge with a lethal dose of NiV Bangladesh and NiV Malaysia, and partially protected against HeV challenge. Furthermore, antibodies elicited by vaccination alone provided partial protection against a NiV Bangladesh challenge.

Two genetic lineages of NiV have been described; NiV Malaysia and NiV Bangladesh [10–12]. Although NiV Malaysia has not caused an outbreak in humans since 1999, the virus was isolated from *Pteropus vampyrus*, *Pteropus hypomelanus* and *Pteropus lylei* in Malaysia and Cambodia [43–45] and another spillover event could occur. Having one vaccine that protects against both lineages of NiV virus would be the easiest and cheapest countermeasure. A single-dose vaccination with ChAdOx1 NiV_B_, which is based on NiV Bangladesh, fully protected Syrian hamsters against lethal disease caused by NiV Malaysia. The G proteins of the NiV strains used in this study are 95.5% pairwise identical on the amino acid level, with 27 amino acid differences scattered throughout the protein. Although we did not see sterile protection against NiV Malaysia, none of the vaccinated animals showed signs of disease and all were protected against lethal disease. These results suggest that ChAdOx1 NiV_B_ could protect against both lineages of NiV.

Like NiV, HeV is a species in the *Henipavirus* genus and thus we investigated cross-protection of ChAdOx1 NiV_B_ against a lethal challenge with HeV in Syrian hamsters. The G protein of the HeV strain used in this study was 78.2% identical to the ChAdOx1 NiV_B_ G protein; 133 amino acids differ between the two proteins. ChAdOx1 NiV_B_ only protected partially against HeV challenge; four out of six animals did not survive challenge. We observed a non-significant decrease in infectious HeV titer in lung and brain tissue of vaccinated animals compared to control animals. It is possible that disease progression in vaccinated animals is delayed compared to control animals. This is supported by the delay in time to death; whereas the average time to death is 5 days in control animals, it is 6 days in vaccinated animals.

Cross-protection of NiV or HeV vaccines has been studied by other groups as well. An adeno-associated virus vaccine expressing NiV G protein offered 50% protection against a lethal challenge with HeV in hamsters [46]. In contrast, vaccines based on HeV provide full protection against NiV in the ferret and NHP model [36, 41, 47]. Likewise, high levels of cross-protective antibodies were found in sera from HeV-infected individuals, whereas cross-protective antibodies were limited in NiV-infected individuals [48]. This might be caused by induction of a more robust and cross-reactive immune response by native HeV protein compared to NiV protein, as suggested by Bossart *et al*. [48].

Human cases of HeV are associated with direct contact with infected horses, the intermediate animal host of HeV, and direct contact with bats or their products has not yet been associated with HeV infection in humans [49]. It is therefore likely that prevention of HeV in horses will completely prevent human cases. Currently, a HeV vaccine (Equivac) is available for horses and fully protects against HeV [50]. Furthermore, the total number of human cases that contracted HeV is relatively low at 7 [13]. Thus, the requirement of a human vaccine for HeV is therefore less urgent than that of a NiV vaccine.

Previous work has shown that the humoral immune response to NiV vaccination is sufficient to protect Syrian hamsters against a lethal challenge with NiV [27, 42]. Likewise, administration of a human neutralizing monoclonal antibody (m102.4) provided full protection against both HeV and NiV in multiple animal models [51, 52]. Administration of purified IgG obtained from ChAdOx1 NiV_B_ vaccinated hamsters provided partial protection against NiV challenge. Furthermore, infectious virus could only be detected in the lungs of control animals and not in the lungs of vaccinated animals, and thus as in previous studies, ChAdOx1 NiV_B_-elicited antibodies are able to provide protection against a lethal challenge with NiV. Although we were able to detect NiV G protein-specific antibodies in serum obtained from NiV antibody-treated animals, the reciprocal titer was much lower than that detected in serum from Syrian hamsters after a single dose of ChAdOx1 NiV_B_. It is possible that administering a higher dose of IgG would have led to uniform protection.

Two animals treated with IgG purified from animals which received injections with ChAdOx1 FGP survived a lethal challenge with NiV Bangladesh. Occasional survival has been observed in the Syrian hamster model [33]. The increased survival rate might however also reflect a non-specific effect of treatment with IgG, which has been reported previously [53]. As the survival rate was significantly different between the NiV IgG-treated group and the control IgG-treated group, the passive transfer experiment shows that antibodies elicited by ChAdOx1-NiV_B_ are sufficient for protection against a lethal challenge with NiV.

Animals in the passive transfer experiment were observed for 56 days, to ensure that the two animals that survived would not succumb to disease after 28 days.

The Syrian hamster is a suitable initial small animal model to investigate the efficacy of NiV vaccines, followed by the African green monkey [54]. The immune system of African green monkeys is more like humans than that of hamsters and is therefore seen as a more relevant animal model to test NiV vaccines. Based on the results presented in the current manuscript, future studies are planned to test ChAdOx1 NiV_B_ in African green monkeys, supported by the Coalition for Epidemic Preparedness Innovations (CEPI).

We show that ChAdOx1 NiV_B_ provides complete protection against lethal disease in Syrian hamsters challenged with NiV Bangladesh. Furthermore, ChAdOx1 NiV_B_ vaccination results in complete survival but with limited evidence of viral replication after NiV Malaysia challenge, and partial protection against HeV. Passive transfer of antibodies elicited by ChAdOx1 NiV_B_ vaccination provide partial protection against lethal challenge with NiV Bangladesh.
